# The Evolution of Methotrexate as a Treatment for Ectopic Pregnancy and Gestational Trophoblastic Neoplasia: A Review

**DOI:** 10.5402/2012/637094

**Published:** 2012-02-19

**Authors:** Monika M. Skubisz, Stephen Tong

**Affiliations:** ^1^Translational Obstetrics Group, Department of Obstetrics and Gynaecology, University of Melbourne, Mercy Hospital for Women, Heidelberg, 3084 VIC, Australia; ^2^Department of Obstetrics & Gynaecology, Monash University, Level 5, Monash Medical Centre, 246 Clayton Road, Clayton, 3168 VIC, Australia

## Abstract

Methotrexate was developed in 1949 as a synthetic folic acid analogue to compete with folic acid and thus interfere with cell replication. While initially developed as a potential treatment for acute lymphoblastic leukaemia, a serendipitous observation led to methotrexate's use to effect the dramatic cure of a case of advanced choriocarcinoma. This prompted the exploration for the potential of methotrexate to treat other conditions involving disordered trophoblastic tissue. Methotrexate has subsequently revolutionized the treatment of two pregnancy-related conditions—gestational trophoblastic neoplasia and ectopic pregnancy. This article reviews the development of modern treatment protocols that use methotrexate to medically treat these two important gynaecological conditions.

## 1. Introduction

Methotrexate is an antifolate drug which inhibits cell division by interfering with DNA replication [1]. It is used clinically in medicine to treat a range of cancerous and noncancerous conditions. Methotrexate is currently used in gynaecology to treat disorders arising from trophoblastic tissue, namely, ectopic pregnancy and gestational trophoblastic disease. Whilst the incidences of these conditions in pregnancy are relatively rare (ranging from 0.7% for GTD [2] to 1-2% for ectopic pregnancy [3]), their impact on the lives of young women of reproductive age, both in terms of mortality and morbidity (especially loss of reproductive potential), are significant. Methotrexate has contributed to alleviating some of the disease burden of ectopic pregnancy, where it affords approximately 25% of women a nonsurgical and fertility-preserving treatment option [4]. Methotrexate has dramatically improved survival outcomes in gestational trophoblastic neoplasia, where surgery is now only occasionally used as an adjunct to treatment [5]. 

This article reviews methotrexate and its mechanism of action, gestational trophoblastic neoplasia and ectopic pregnancy, and how modern treatment protocols using methotrexate to medically treat these two conditions developed.

## 2. Methotrexate

### 2.1. History and Origins

Methotrexate was one of the first drugs synthesized for a specific chemotherapeutic purpose—*in situ* folic acid inhibition for the treatment of acute lymphoblastic leukaemia (ALL) in children [1]. Its history is closely related to the discovery and characterisation of folic acid [6]. The “factor” later shown to be folic acid was discovered by missionary physician Lucy Wills in India in the 1930s, when she used Marmite to treat megaloblastic anaemia in impoverished, pregnant textile workers [7]. In the 1940s, this “factor” was isolated from spinach leaves and called folic acid (*folium* being Latin for leaf) [7]. At this time, American pathologist/pediatrician Sidney Farber had noted the morphological similarities between ALL and megaloblastic anaemia and trialled folic acid in the treatment of children with ALL; however, it paradoxically accelerated disease progression [1]. Folic acid-deficient diets were subsequently shown to decrease the leukaemia cell count, and this led to the development of folic acid antagonists [1]. Methotrexate was the second drug to be developed for this purpose by a group of researchers at the Lederle Laboratories in 1949 [1, 7]. Methotrexate induced remission of ALL in children but did not cure the disease [6]. Its application to the treatment of a woman with terminal choriocarcinoma in 1956 produced the first cure of a solid tumour by chemotherapy [6], and its use in the treatment of GTN and ectopic pregnancy expanded from there [8].

### 2.2. Mechanism of Action

**Figure 1:**
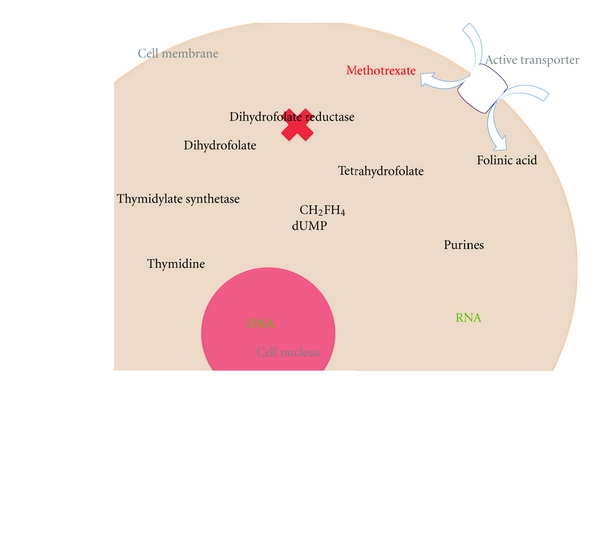
The mechanism by which methotrexate inhibits cellular proliferation. Active transporter includes the reduced folate carrier and an endocytic pathway activated by a folate receptor; dUMP: deoxyuridine monophosphate; CH_2_FH_4_: methylenetetrahydrofolate.

Methotrexate was created to compete with folic acid for biological activity in the body. It does so by competitively binding with the enzyme dihydrofolate reductase with much greater affinity than folic acid [9]. This prevents the conversion of dihydrofolate to tetrahydrofolate, which is essential in the de novo synthesis of purine nucleotides and thymidylate, which are themselves essential substrates of DNA synthesis, repair, and cell proliferation [1, 10] (Figure 1). Methotrexate thus blocks cell proliferation in the S phase of the cell cycle during which DNA replication occurs, making rapidly dividing cells such as trophoblast especially susceptible to its action [1, 10]. It also means that methotrexate has a nonspecific mechanism of action, and with this comes the potential for numerous side effects.

### 2.3. Clinical Use and Side Effects

Methotrexate has a wide range of indications that extend beyond its original design for the treatment of hematological malignancy. It is still used for the treatment of neoplasms such as leukaemias and lymphomas, but also lung cancers, breast cancer, head and neck cancers, osteosarcomas, bladder cancer, and GTN [1, 11]. It has been found to have an immunomodulatory effect in autoimmune conditions such as rheumatoid arthritis, although its mechanism of action here remains unknown, and psoriasis, where it is thought to impede the rapid turnover of skin cells characteristic of the condition [1, 11], and is used occasionally in Crohn's disease, multiple sclerosis, and psoriatic arthritis [11]. It is also used in the treatment of ectopic pregnancies by extension of its use in GTN [8, 11].

Owing to its nonspecific actions on cell division, methotrexate is associated with toxicities and side effects that include every organ system [11]. The exact likelihood and incidences vary slightly depending on the condition being treated, the route of administration, the dosage, and length of treatment [11]. By its mechanism of action methotrexate preferentially targets rapidly dividing cells; therefore, the hematological, gastrointestinal, and dermatological systems which feature a high turnover of cells are the most likely to display signs and symptoms of toxicity such as neutropenia and generalized myelosuppression, nausea, vomiting, diarrhoea, and gastrointestinal inflammation as well as generalized erythema, rash, photosensitivity, and alopecia [11]. These side effects have been greatly ameliorated in the field of gynaecology by the development of a low, single-dose protocol in the treatment of ectopic pregnancies [12], and to some extent with the use of folinic acid (a form of tetrahydrofolate) rescue in GTN chemotherapy protocols [8, 11, 13].

## 3. Gestational Trophoblastic Neoplasia

### 3.1. History and Definition

Gestational trophoblastic disease (GTD) consists of a range of premalignant and malignant tissues that arise from trophoblastic cells in association with any type of gestational event. The premalignant forms of the disease are called molar pregnancies and comprise over 90% of GTD [14]. Molar pregnancies consist of abnormally proliferative trophoblastic tissue and occur after an abnormal fertilization event. There are two types of molar pregnancies: complete hydatidiform mole (CHM), where there is no maternal chromosomal material in the ovum and either one sperm fertilizes the ovum and replicates its DNA (androgenetic monospermic-90%) or two sperm fertilize the empty ovum (androgenetic dispermic-10%), and partial hydatidiform mole (PHM) where an apparently normal ovum with maternal chromosomal material is fertilized by two sperm resulting in triple the normal amount of chromosomal material (biparental triploid) [5]. A rare form of biparental CHM is associated with an autosomal recessive mutation on chromosome 19q (*NLRP7)* and is thought responsible for recurrent molar pregnancy [5]. Malignant forms of GTD mainly consist of invasive mole and choriocarcinoma, and occasionally the rare placental-site trophoblastic tumour (PSTT). These malignant forms of GTD are called gestational trophoblastic neoplasias (GTN).

Hippocrates himself was most likely referring to GTD when he described “dropsy” of the uterus around 400BC [5]. It wasn't until 1895 that an association between pregnancy and GTD was confirmed when Marchand determined that the tumours arose from fetal cell lines [5, 15]. Prior to the 1950s and the era of chemotherapy, choriocarcinoma, the most aggressive form of GTN, was fatal in 90–95% of cases where metastases were present [15]. In 1956, the antifolate drug methotrexate (see above) was given to a young woman with advanced metastatic choriocarcinoma who was expected to die within a matter of months. The idea to test methotrexate for this condition came from an earlier observation where methotrexate given to a patient with malignant melanoma unexpectedly eradicated the presence of *β*hCG from her urine [6]. This terminally ill woman with choriocarcinoma not only got better with methotrexate treatment but was discharged home four months later with no identifiable disease [6]. This was the first cure of any solid tumour with chemotherapy [6].

### 3.2. Incidence, Risk Factors, and Mortality

The incidence of molar pregnancies is notoriously difficult to determine, as the diagnosis relies on highly specialized histopathological examination [16]. Furthermore, estimates of the incidence of molar pregnancies are calculated against either total number of pregnancies, deliveries, or both, creating confusion, and these statistics themselves are prone to underreporting [16]. Estimates of the incidence of molar pregnancy in the Western world including Australia are 0.5–1/1000 pregnancies, whilst the much higher incidence in Asia (1-2/1000 pregnancies in Japan and China and up to 12/1000 pregnancies in Indonesia) is believed to be genuinely reflective [16].

Extremes of maternal age and previous molar pregnancy are the two most strongly proven risk factors [14]. Pregnancies occurring during teenage years carry a 1.5–2-fold increased risk of molar pregnancy, whereas, in pregnancies occurring when a woman is 40-year old or more, the risk is increased 5–7.5-fold [5, 16]. Similarly, if a woman has had one previous molar pregnancy, her risk of recurrence in a future pregnancy is 1% compared to 0.1% of the general population, and if she has had two or more, this risk rises to between 16 and 28% [14, 16]. Recurrent disease may be due to the aforementioned genetic mutation (*NLRP7)*. Other risk factors such as maternal blood group, smoking, parity, and oral contraceptive pill use have not been consistently proven [14]; however, the worldwide distribution of areas with high levels of vitamin A (beta carotene) deficiency, which causes abnormal spermatogenesis and spontaneous abortion in Rhesus monkeys, correlates with areas with a high incidence of molar pregnancy [5, 14].

Molar pregnancies have malignant potential, with local invasion a feature in 15% of cases and/or metastases in 4% [17]. Complete moles have a higher rate of persistence and neoplastic potential at 6–30% of cases, compared to 0.5–3% after partial mole [5, 14, 18]. Risk factors for developing invasive, persistent disease are: serum *β*hCG levels >100,000 IU/L at diagnosis, associated large ovarian theca lutein cysts (>6 cm), uterine size larger than expected for dates, and maternal age >35 y.o. [19]. Choriocarcinoma and PSTT, the other forms of GTN, occur more frequently after nonmolar pregnancies at a rate of between 1 : 20,000 and 50,000 pregnancies [5, 20]. Approximately 25% of cases, however, still occur after complete molar pregnancy [20], and molar pregnancies precede 90% of cases of GTN [14].

The advent of chemotherapy changed metastatic GTN from a fatal disease in 90–95% of cases [15] to being curable in 85% of cases [5, 20]. For women with low-risk (see below) GTN, the cure rate approaches 100% [20]. However, despite a good 5-year-survival rate of 89.5% [21], there is scope for further improvement in the management of GTN. Mortality from choriocarcinoma after nonmolar pregnancy is 21% compared to 6% after molar pregnancy and is mainly due to late diagnosis when the disease is already advanced [22]. Deaths still occur from drug resistant disease. Cost-effective screening or surveillance measures to identify women with GTN after nonmolar pregnancies would potentially help to further improve survival by diagnosing the condition earlier in this cohort. Newer agents with greater efficacy and lesser toxicity could greatly improve the care of women with this condition [5, 22].

### 3.3. Management of Molar Pregnancy

First-line treatment for molar pregnancies is suction curettage, and indeed the majority of molar pregnancies are diagnosed after histopathology testing of products of conception from this procedure [23]. This is because GTD presents similarly to and is frequently misdiagnosed as incomplete/missed miscarriages, and even ectopic pregnancy. Once the products of conception have been removed surgically and the pathological diagnosis confirmed as either complete or partial molar pregnancy, patients are followed up with serial serum and/or urinary *β*hCG levels until they normalize (<5 IU/L) [24, 25]. 98% of women who develop GTN (invasive mole) will be identified this way within the first 6 months after surgical evacuation [26]. Serum/urinary *β*hCG levels should be measured at least every 2 weeks [5, 20], and women whose *β*hCG normalizes within 56 days have a reduced risk of developing GTN [5]. They are followed up for a further 6 months, and if their *β*hCG levels remain normal, they are then discharged from surveillance. Of the women whose *β*hCG took longer than 56 days to normalize, a study by Sebire et al. showed that extended surveillance for 2 years picked up only 1 additional woman who went on to develop GTN and required treatment. Hence since 2007, women whose *β*hCG takes longer than 56 days to normalize are followed up for 6 months from the time of *β*hCG normalization [26]. A total of 3 of 6701 women (<0.05%) who went on to develop GTN were missed by this surveillance protocol and represented with clinical symptoms [26].

### 3.4. Management of GTN

If hCG followup for GTD detects a persistence or rise in levels, invasive and therefore malignant forms of GTD, called gestational trophoblastic neoplasias (GTN), are assumed [27]. The Federation of Gynaecologists and Obstetricians proposed the following consensus diagnostic criteria of GTN using *β*hCG surveillance: (1) a *β*hCG level plateau of four values ±10% over 3 weeks, (2) a *β*hCG level increase of more than 10% of three values over 2 weeks, and (3) persistence of detectable *β*hCG for more than 6 months after curettage [20]. GTN can, however, occur after any form of pregnancy; approximately 50% follow a normal pregnancy, 25% after molar pregnancy, and the remaining 25% after other gestational events such as ectopic pregnancies and miscarriages [20]. Those occurring after a nonmolar pregnancy generally present later and with more advanced disease as they are not identified through *β*hCG surveillance but through clinical presentation and often carry a worse prognosis as a result [22].

Repeat suction curettage is generally avoided. It carries high risk of uterine perforation and in particular, significant haemorrhage, as trophoblastic tissue is highly vascular and prone to arteriovenous malformations in GTN [28]. More than 50% of GTN patients will still need chemotherapy after a repeat curettage; therefore, chemotherapy is the preferred first-line treatment [5]. For women who have completed their families, hysterectomy is an option to treat GTN apparently confined to the uterus; however, because of GTNs ability to micrometastasise very early, this does not obviate the need for *β*hCG surveillance or the possibility of requiring chemotherapy [5].

The form of chemotherapy (single agent versus multiple agents) is determined by the Modified WHO Prognostic Index Score (PIS) of the patient [5, 20] (Table 1). Unlike other cancers where the staging (i.e., anatomical spread) of the tumour best determines management and correlates to prognosis, it is the presence of certain risk factors that has been shown to correlate most highly with treatment outcome and prognosis in GTN [29]. Low-risk assessment indicates that the patient is likely to respond favourably to single-agent chemotherapy, and high-risk patients require more aggressive, multiagent chemotherapy to achieve disease remission [29]. The FIGO staging of GTN is still used to describe anatomical spread of the disease [29] (Table 2).

Low-risk disease is defined as a PIS of 0–6 and represents approximately 95% of GTN patients [5]. These women are highly likely to respond to single-agent chemotherapy with either methotrexate or actinomycin D (a very old antibiotic with anticancer activity through inhibition of DNA replication), the two most widely used first-line agents [31]. Various methotrexate regimens are used, with little evidence of the superiority of one regimen over the other [29]. The remission rate of methotrexate therapy ranges from 50 to 90% depending on the route, dose, frequency of administration, and patient selection criteria used [5]. Some studies suggest that actinomycin D is more likely to induce remission than methotrexate [5]; however, it also seems more likely to cause toxicity such as alopecia [5, 29]. An 8-day multidose methotrexate protocol with intervening folinic acid (to minimize toxicity) was first suggested by Bagshawe and Wilde in 1964 [32], and this regimen is the first-line treatment for low-risk disease used by large centres for trophoblastic disease management and research in both the US and the UK, and hence the most widely used regimen in the world [5, 31, 33]. It achieves remission in 90% of low-risk stage I patients and 70% of low-risk stage II-III patients [29] and is associated with low toxicity—<15% patients experience nausea, <5% vomiting, and approximately 2% develop mouth ulcers, sore eyes or chest or abdominal pain from pleuritic or peritoneal serositis [5, 29]. If first-line treatment fails, treatment with the alternate first-line agent (methotrexate or actinomycin D) or even multiagent chemotherapy are used to attain an overall survival rate of nearly 100% [5]. Treatment is continued until the serum *β*hCG normalizes (<5 IU/L) for at least three consecutive weeks [31]. 

Multiagent chemotherapy is used to treat high-risk GTN, defined as stage IV disease or stage II-III disease with a PIS of 7 or above, as well as PSTT and treatment-refractive low-risk disease [29]. Again, a wide variety of regimens are employed worldwide, with very little evidence to show superiority of any one regimen. The most widely used multiagent chemotherapy regimen EMA-CO-consists of: etoposide, methotrexate, actinomycin D, cyclophosphamide, and vincristine (Oncovin). It achieves a 5-year-survival rate of 86.5% [29, 34] and is a relatively well-tolerated regimen, with alopecia being the commonest side-effect, high-grade haematologic toxicities experienced by less than 2% of patients, and more than half of patients retaining their fertility [29]. There are no randomized controlled clinical trials comparing EMA-CO to other combination chemotherapies [34]. Other combination therapies include EMA (etoposide, methotrexate, and actinomycin D) with one retrospective study suggesting similar efficacy but inconclusively less toxicity compared to EMA-CO, and MAC (methotrexate, actinomycin D, and chlorambucil) which in a retrospective study has much lower durable remission rates compared to EMA-CO and requires a greater number of cycles to achieve remission [29]. 

Methotrexate has revolutionized the treatment of GTN. Prior to 1956, women with what we now call low-risk disease would have frequently lost their fertility through hysterectomy, which was the mainstay of treatment. Those with high-risk disease inevitably died from the condition. Methotrexate not only replaced surgery as a treatment option, to this day it is used to cure almost 100% of women with low-risk disease and up to 86% of women with high-risk disease in combination with newer chemotherapeutics. 

## 4. Ectopic Pregnancy

### 4.1. Definition and History

An ectopic pregnancy is one that implants outside of the uterus, in one of the Fallopian tubes in 95.5% of cases [35], but potentially anywhere in the abdominal and pelvic cavities. The name is derived from the Greek word *ektopos*, meaning “out of place” [36], and the condition has been described in medical literature since the 11th century when the Arabic physician Abulcasis extracted a fetus from a swelling he drained through the abdominal wall [37]. It was considered a “universally fatal accident” until the 19th century, when attempts such as “vaginal section,” maternal starvation, purging, bleeding, strychnine administration, and fetal morphine injection “improved” the survival prognosis to between 72 and 99% [37]. 

### 4.2. Significance, Risk Factors, Incidence, and Mortality

The incidence of ectopic pregnancies is between 1-2% of all pregnancies [3, 38]. Recognized risk factors fall into two categories: contraceptive failure, where an intrauterine device has not prevented fertilisation and is associated with a 3-4% risk of ectopic pregnancy [39], and reproductive failure, where risk factors include a history of pelvic inflammatory disease (especially Chlamydia trachomatis [40]), tubal damage from other causes such as previous surgery, previous ectopic pregnancies, smoking, advancing maternal age, infertility, and assisted reproductive techniques [41, 42]. 

Ectopic pregnancy is still a potentially life-threatening condition, as the invasive and angiogenic nature of trophoblastic tissue outside of the specialized lining of the uterus means that the growing pregnancy can disrupt maternal vasculature and cause fatal haemorrhage. The advent of modern surgical techniques, anaesthesia, blood transfusions, and antibiotics in the early 20th century, and more recently ultrasound and medical treatment for the condition in the late 20th century, have seen the mortality of ectopic pregnancy fall to 0.5 deaths per 100,000 live births in the US, 0.47 per 100,000 maternities in the UK, and 0.13 per 100,000 births in Australia [38, 43, 44]. 

### 4.3. Surgical Management

Ectopic pregnancies were increasingly described in 17th and 18th century France, either at autopsy or during abdominal surgeries [37, 45]. In America, the first abdominal surgery for ectopic pregnancy was performed in 1759 by John Bard, and surgical management became increasingly attempted in the 19th century [45]. The survival rate of women who were operated on in the 1800s, however, was 5 in 30, worse than the 1 in 3 who survived with no intervention [45]. Robert Lawson Tait, an eminent British surgeon, described treatment of ruptured ectopic pregnancy by ligating bleeding vessels at laparotomy in 1884. This was a major advance in developing effective surgical management of this condition [45]. 

As operative techniques have developed and improved, laparoscopy has replaced laparotomy as the preferred approach, mainly due to the significant cost savings it confers. This is a result of shorter operating time, less intraoperative blood loss, shorter hospital stay and shorter convalescence associated with laparoscopy as opposed to laparotomy [46]. These factors make laparoscopy much more acceptable to patients as well. However, systematic review suggests that the laparoscopic approach is significantly less successful than laparotomy in eliminating ectopic pregnancy (OR 0.28, 95% CI 0.09–0.86), mainly due to a higher incidence of persistent trophoblastic tissue after salpingostomy (OR 3.5, 95% CI 1.1–11) [47]. 

Two techniques are described to remove the ectopic pregnancy from the Fallopian tube *salpingectomy*, where the pregnancy is removed *en bloc* with the tube and *salpingostomy*, where an incision is made on the Fallopian tube over the swelling, the ectopic pregnancy carefully removed with forceps or irrigation and the incision either closed or left to heal by secondary intention [45]. There are no prospective studies comparing subsequent fertility rates after either salpingectomy or salpingostomy, however, retrospective studies suggest no significant difference [48]. Salpingostomy is, however, associated with a 5–8% risk of persistent trophoblastic tissue and is less cost effective as a result of the subsequent monitoring and treatment that this necessitates [48]. Salpingectomy is considered preferable when there is significant haemorrhage and/or damage to the tube, when ectopic pregnancy has recurred in the same Fallopian tube and when future pregnancies are not desired [45]. A prospective, randomized control trial is currently underway to evaluate whether one technique is better than the other in the management of women with ectopic pregnancies and healthy contralateral tubes who wish to conceive in future [49]. In the meantime, preference is largely region and operator dependent. 

### 4.4. Medical Management

Extirpative surgery remained the only effective medical intervention for ectopic pregnancy until medical management was (re-)introduced in the 1980s [8]. Methotrexate was first used in diagnosed ectopic pregnancies in the 1960s to aide safe surgical removal of the placenta from its abdominal implantation sites in second- and third-trimester cases [50]. In the 1980s, the use of methotrexate for treatment of ectopic pregnancies was based on its use in GTN, that is, fixed, multidose regimens with intervening folinic acid rescue [13, 51]. These were full chemotherapeutic doses, which although achieving cure, also produced significant side effects in women. The potential of methotrexate/folinic acid therapy as an outpatient treatment for ectopic pregnancy was first explored by Stovall et al. in 1989 [12]. At this time, however, the gold standard of diagnosis of ectopic pregnancy was by direct visualization at surgery, as ultrasound was still too crude an instrument to rely upon in this potentially life-threatening situation [52]. Surgery as part of the diagnostic logarithm of ectopic pregnancy heavily negated the benefits of then proceeding to medical management, both in terms of cost effectiveness and acceptability to patients [8, 46]. 

Transvaginal ultrasonography (TVUS) became increasingly available in the late 1980s, and by the mid 1990s sensitivity and specificity were calculated at 84.4 and 98.9%, respectively, [53]. This rapid technological advancement and improvement in image quality meant that, by the mid to late 1990s, TVUS became the preferred means of diagnosing ectopic pregnancy although laparoscopic visualization remains the gold standard for diagnosis [52]. 

Stovall et al. remained the pioneers of outpatient treatment of ectopic pregnancies with methotrexate and undertook much of the research that underpins the current “single-dose” regimen and monitoring protocol used worldwide today [54]. Their first trial with the aim of using a single dose of methotrexate to treat ectopic pregnancy was published in 1991, with a cure rate of 96.7% [55]. They included TVUS in a nonsurgical workup logarithm of patients with suspected ectopic pregnancies from 1993 [56] and developed a monitoring protocol which is based on serial serum *β*hCGs taken on day 1, 4, 7, and weekly until resolution. Efficacy of treatment is determined when there is a ≥15% fall in serum *β*hCG between day 4 and day 7. This definition of treatment success has a positive predictive value (PPV) of 93%, with a sensitivity of 93% and a specificity of 84.2% [57]. If there is not a ≥15% fall in serum *β*hCG between day 4 and day 7, a second (and third) intramuscular injection of methotrexate (50 mg/m^2^) can be given. Approximately 20% of patients will require more than one dose of methotrexate to achieve *β*hCG normalization [54]. 

The use of methotrexate in the treatment of ectopic pregnancy is limited by its efficacy, which drops sharply from 96% at *β*hCG serum levels between 2000 and 4999 IU/L, to 86% for serum *β*hCG levels between 5000 and 9999 IU/L (OR 3.76, 95% CI 1.16–12.33)[58]. This necessarily limits the number of women with ectopic pregnancies eligible for medical management at the time of diagnosis. 

Methotrexate has been combined with mifepristone (RU486), a progesterone antagonist, in the treatment of stable ectopic pregnancies. Two trials have shown that single-dose methotrexate was slightly less successful at curing ectopic pregnancy than when 600 mcg of mifepristone was added orally (OR 0.84, 95% CI 0.71–1.0) [59]. In addition to the borderline significance, van Mello notes that the cohorts of women featured relatively low starting serum *β*hCGs (between 346 and 1,679 IU/L), *β*hCG itself being the strongest prognostic indicator of medical treatment success [59, 60]. 

Other medical treatments such as prostaglandins and/or hyperosmolar glucose have been compared to treatment with systemic methotrexate. Prostaglandins alone or in combination with hyperosmolar glucose showed no difference in treatment success or side effects compared to methotrexate alone, and a study examining methotrexate versus hyperosmolar glucose was abandoned due to a high failure rate in the latter group (OR 0.30, 95% CI 0.05–2.0) [47]. Alternative means of administering methotrexate have also been studied, for example direct injection into the tubal mass at laparoscopy or with ultrasound guidance trans-vaginally. Both methods require clinicians with a higher skill set than that required for systemic administration of methotrexate and are less successful treatments than laparoscopic salpingectomy [47]. Hence, systemic methotrexate, particularly the “single-dose” regimen, remains the only medical treatment of ectopic pregnancy in common clinical use. 

Most centres use a protocol to carefully select which women diagnosed with ectopic pregnancies are suitable for medical outpatient treatment with “single-dose” systemic methotrexate. This is determined primarily by the haemodynamically stable condition of the woman and lack of clinical evidence of ectopic pregnancy rupture: blood pressure, pulse rate and oxygen saturation within normal limits, no evidence of guarding or rigidity on abdominal examination, normal red blood cell indices on blood tests, and minimal or no evidence of blood in the Pouch of Douglas on TVUS. Additionally, determinants of likelihood of medical treatment success are assessed when deciding on whether a woman is suitable for medical treatment of her ectopic pregnancy. They generally include: a serum *β*hCG with an upper limit between 3000–5000 IU/L, a gestational sac size of less than 3-4 cm, and no fetal heart visualized on TVUS. 

### 4.5. Surgery versus Medical Treatment

Systemic methotrexate has been compared to laparoscopic salpingostomy as a treatment for tubal ectopic pregnancy. Multidose methotrexate was comparable to laparoscopic salpingostomy in terms of treatment success in women with ectopic pregnancies of any size or starting serum *β*hCG level (OR 1.8, 95% CI 0.73–4.6); however, it was associated with significantly greater incidence of side effects (60% versus 12%) and poorer health-related quality of life (*P* < 0.05) [47]. Multidose methotrexate was also a more expensive treatment option [47]. Single-dose systemic methotrexate compared to laparoscopic salpingostomy in women with selected ectopic pregnancies (less than 4 cm on TVUS and with starting serum *β*hCGs less than 10,000 IU/L) was a far less effective treatment for ectopic pregnancy (OR 0.38, 95% CI 0.20–0.71), however, when allowing for additional doses of methotrexate if serum *β*hCG was falling inadequately, there was no difference in treatment success compared with laparoscopic salpingostomy (OR 1.1, 95% CI 0.52–2.3) [47]. 

Methotrexate therefore has an established and well-proven role in the treatment of ectopic pregnancy, and although its use is limited by serum *β*hCG level and to a lesser extent, ectopic pregnancy size, it nevertheless confers cost, fertility, and arguably improved treatment-related quality of life and recovery benefits to approximately one quarter of women with ectopic pregnancies.

## Figures and Tables

**Table 1:** Modified WHO prognostic scoring system as adapted by FIGO [30].

Scores	0	1	2	4
Age	<40	>40	—	—
Antecedent pregnancy	Mole	Miscarriage	Term	—
Interval months from index pregnancy	<4	4–6	7–12	>12
Pretreatment serum *β*hCG (IU/L)	<10^3^	10^3^–10^4^	10^4^–10^5^	>10^5^
Largest tumour size (including uterus) (cm)	<3	3-4	≥5	—
Site of metastases	Lung	Spleen/kidney	Gastrointestinal	Liver/brain
Number of metastases	—	1–4	5–8	>8
Previous failed chemotherapy	—	—	Single drug	≥2 drugs

**Table 2:** GTN FIGO staging and classification [30].

Stage I	Disease confined to the uterus
Stage II	GTN extends outside of the uterus but is limited to genital structures (adnexa, vagina, broad ligament)

Stage III	GTN extends to the lungs, without genital tract involvement

Stage IV	All other metastatic sites
